# Clinically Important Age-Related Differences in Sleep Related Disordered Breathing in Infants and Children with Prader-Willi Syndrome

**DOI:** 10.1371/journal.pone.0101012

**Published:** 2014-06-30

**Authors:** Michal Cohen, Jill Hamilton, Indra Narang

**Affiliations:** 1 Division of Endocrinology, The Hospital for Sick Children, Toronto, Ontario, Canada; 2 The University of Toronto, Toronto, Ontario, Canada; 3 Division of Respiratory Medicine, The Hospital for Sick Children, Toronto, Ontario, Canada; University of Adelaide, Australia

## Abstract

**Background:**

Sleep related disordered breathing (SDB) in pediatric Prader-Willi Syndrome is gaining increased attention due to the possible association of growth hormone therapy, SDB and sudden death. However data on the patterns of SDB and their management, particularly in infants in this population, is lacking.

**Objective:**

The aim of this study was to 1) describe patterns of SDB in growth hormone naive infants with PWS and the management of these disorders in our institution 2) Compare the patterns of sleep disorders between infants and children with PWS.

**Methods and Design:**

Polysomnograms of infants and children (0–18 years of age) with Prader-Willi Syndrome were reviewed. Age, sex, anthropometrics, sleep architecture, obstructive and central apnea indices and oxygen saturations were recorded. Data of infants with central sleep apnea treated with oxygen were analyzed to evaluate the efficacy of this treatment. The main outcome measures were obstructive and central apnea indices on a polysomnogram.

**Results:**

Data of 44 patients, 23 under 2 years of age and 21 older children were included. Infants when compared with older children were more likely to experience central sleep apnea (43% vs. 5%; p = 0.003). In older children obstructive was significantly more prevalent than central sleep apnea. Supplemental oxygen was used to treat 9/23 infants with central sleep apnea. Oxygen therapy resulted in a significant decrease in the median central apnea index from 14 (5,68) to 1 (0,6; p = 0.008) events/hour and an improvement in the oxygen saturation nadir from 70% (52, 92) to 81% (64, 95; p = 0.080).

**Conclusions:**

Central sleep apnea with associated oxygen desaturations is more prevalent in infants compared with older children with Prader-Willi Syndrome. Supplemental oxygen was efficacious in treating central sleep apnea in infants. Routine sleep surveillance for all children with Prader-Willi Syndrome and treatment with oxygen for central sleep apnea should be considered.

## Introduction

Prader-Willi syndrome (PWS) is a complex genetic disorder that occurs in 1/10,000 to 1/25,000 live births. PWS is characterized by severe hypotonia, poor feeding, hypogonadism, failure to thrive in early infancy with hyperphagia, obesity, short stature and cognitive and behavioral disturbances appearing later in childhood [1]. Sleep related disordered breathing (SDB), a group of respiratory disorders specific to or exacerbated by sleep, have been reported in PWS [2]. SDB includes obstructive sleep apnea (OSA), central sleep apnea (CSA), and hypoventilation syndromes. OSA is considered the most common form of SDB in children, with adenotonsillar hypertrophy as the usual etiologic factor. Sleep problems reported in children with PWS include not only OSA and CSA but also excessive daytime sleepiness, altered sleep architecture and abnormal arousal and cardiorespiratory response to hypoxia and hypercapnia [2]–[5]. More recently, sleep disorders in PWS, particularly OSA in the obese older children have gained increasing attention due to the possible association of growth hormone (GH) therapy, abnormal sleep related breathing and sudden death [6], [7]. However to date few studies have focused on the prevalence, severity, nature and management of sleep disorders in infants with PWS and none have specifically compared these with older children [8]–[10]. The benefits of GH on improving lean muscle mass and its likely impact in younger, very hypotonic children has led to an increase in the prescription of GH in infants with PWS with a corresponding increase in sleep surveillance. The aim of this study was to 1) describe the specific patterns of SDB in GH naive infants with PWS and the management of these disorders in our institution, and 2) Compare the patterns of sleep disorders between infants and children with PWS thus providing data on the course of SDB in this population.

## Methods

### Data collection

Data were collected from the Sleep database at the Hospital for Sick Children, Toronto. All children 0–18 years of age with PWS who had a GH naive baseline polysomnography (PSG) performed between January 2005 and June 2013, were included in this evaluation. The most common indication for referral was evaluation prior to commencing GH therapy. The wide age range reflects the age of referral to our program. Exclusion criteria: (i) patients already treated for OSA (ii) additional diagnoses or medications that might affect the occurrence of SDB (eg. cardiac anomalies). Data collected included sex, medical diagnoses, age, height and weight at the time of the PSG and PSG variables as outlined below. Body mass index (BMI) was calculated as weight(kg)/height(m)^2^. BMI z-scores were calculated according to age- and sex-specific growth curves of the World Health Organization in which overweight is defined as a BMI z-score between 1–2 and obesity as a BMI z-score ≥2 [11].

### Polysomnography studies

All PSG studies were overseen and reported by pediatric sleep physicians at the Hospital. Patients underwent standard overnight PSG using a XLTEK data acquisition and analysis system (Natus Medical, San Carlos, California). PSG measurements included electroencephalography, electrooculography, and submental and bilateral anterior tibialis electromyography. Respiratory measurements included chest wall and abdominal movements recorded by belts, nasal airflow using a nasal air pressure transducer and nasal thermal sensor, oxygen saturation (SpO_2_), and transcutaneous carbon dioxide (CO_2_). Information obtained from PSG included sleep onset latency, REM latency, total sleep time, sleep efficiency, time spent in each sleep stage (N1-3 and rapid eye movement (REM)) and snoring. Respiratory data included counts and indices of obstructive apneas, obstructive hypopneas, central apneas and mixed apneas. All events were scored in accordance with the American Academy of Sleep Medicine scoring guidelines [12], studies performed before 2007 were rescored according to these guidelines. An obstructive apnea was scored when airflow dropped by more than 90% from baseline for at least 90% of the entire respiratory event with chest and/or abdominal motion throughout the entire event, for the duration of at least 2 baseline breaths. An obstructive hypopnea was scored when airflow dropped at least 50% from baseline for a duration of at least 2 baseline breaths, accompanied by a minimum 3% drop in SpO_2_, arousal, or awakening. A central apnea was defined as cessation of airflow with the absence of respiratory and abdominal effort for a minimum of 20 seconds or the duration of at least 2 baseline breaths, in which case the event must have been accompanied by a minimum 3% drop in SpO_2_, arousal, or awakening. A mixed apnea was defined as a drop in airflow of more than 90% from baseline for at least 90% of the entire respiratory event, for a duration at a minimum of 2 baseline breaths, associated with absent inspiratory effort in the initial portion of the event, followed by resumption of inspiratory effort before the end of the event. OSA severity was graded according to accepted clinical criteria. The Obstructive Apnea-Hypopnea Index (OAHI) reflects the number of obstructive apneas, mixed apneas, and obstructive hypopneas per hour during sleep. With regards to normative data, Beck and colleagues showed that across a wide age span of children (1–18 years of age), an OAHI index ≤1.4 events/hour and a CSA index ≤1.0 events/hour can be classified as normative PSG values [13]. Although there are limited normative CAI data in very young children, Scholle et al prospectively collected data from PSGs of 209 healthy children [14]. The PSGs were scored according to the AASM guidelines. The CAI was defined as the number of central apneas per hour during sleep. The median (10th–90th percentile) CAI was 2.8 events/hour (1.0–4.3) in children <2 years of age. By age 5 years, the median (10–90^th^ percentile) was 1.1 events per hour (0.5–3.2). The authors also showed that oxygen desaturations 3% or greater were uncommonly associated with central apneas. Our study used the following definitions for OAHI and CAI; an OAHI of <1.5 events/hour was considered normal, an OAHI of >1.5 to <5 events/hour indicated mild OSA, an OAHI of >5 to <10 events/hour indicated moderate OSA, and an OAHI of ≥10 events/hour indicated severe OSA [15]. To avoid misclassification in our study, a CAI ≥5.0 events/hour was considered abnormal [14]. We used the term CSA to define a CAI ≥5events/hour with a minimum nadir of SpO_2_ of 92%. Patients were classified as having 1) OSA, 2) CSA, 3) both OSA and CSA or 4) no OSA or CSA.

### Management of CSA

In our institution, PWS infants with CSA are usually treated with nocturnal supplemental low flow oxygen. Supplemental oxygen is usually trialed during a PSG so the response to oxygen can be evaluated. This is undertaken in 2 ways, 1) a full baseline PSG confirming CSA followed by a second overnight PSG when oxygen is applied or 2) A single spilt night PSG is undertaken where the patient is in room air for the first half of the night followed by supplemental oxygen in the second half of the night. This option is usually for families that are unable to attend for 2 PSGs on 2 nights. PSG variables from a split night study or from 2 separate PSG studies were reviewed for this study.

### Management of OSA

Children with mild OSA were treated with nasal steroids if there was a history of nasal congestion. Children with moderate or severe OSA (>5 events/hour) were referred to ENT for evaluation for adenotonsillar hypertrophy as per standard clinical care [16].

### Statistical analysis

The Statistical Package for Social Sciences software (SPSS statistics version 19.0) was used. Patients were categorized into 2 groups based on age: (i) Infants <2 years of age and (ii) Children 2 years of age and older. The rationale for this age cutoff is that above 2 years of age children with PWS are beginning to develop hyperphagia and risk for increased weight [17]. Additionally although hypotonia is a persistent finding in PWS, the mean age for first walking is at about this age [18], reflecting a transition in the infants ambulatory ability. We hypothesized these differences may lead to distinct patterns of SDB. Baseline characteristics (age, sex and anthropometrics) and PSG study variables were compared between groups; two tailed t-tests or Mann-Whitney U tests were performed for continuous variables and Chi squared tests for categorical variables. To assess the effect of oxygen treatment in infants, Wilcoxon Signed Ranks Tests were performed, comparing sleep variables while breathing room air or with oxygen supplementation. Results were considered statistically significant with p≤0.05.

### Ethics

The study protocol was reviewed and approved by the Research Ethics Board at the Hospital for Sick Children, Toronto, Canada. Due to the retrospective nature of the study, the need for informed consent from the participants/their caregivers for their clinical records to be used in this study, was waived. All patient information was anonymized and de-identified prior to analysis.

## Results

### Subjects

Forty six patients with PWS met inclusion criteria. Of these, 2/46 were excluded, one due to a history of neonatal stroke and one child using continuous positive airway pressure (CPAP) for management of OSA. Data of 44 patients were analyzed (Table 1). The median age was 1.9 years (range 0.3–15.6 years), mean BMI z-score was 1.3±2.1, and mean height z-score was -2.1±1.4. The infants group included 23 patients and the children's group 21 patients. The median ages in the infant and children groups were 1.0 years (range 0.3–1.9 years) and 5.1 years (range 2.4–15.6 years) respectively, mean BMI z-score was 0.1±1.3 and 2.6±2.1 respectively, and mean height z-score was −2.4±1.4 and 1.9±1.4 respectively. A significantly higher proportion of older children with PWS were overweight or obese (17/21 children), when compared with infants (6/23 infants; p<0.001). Upon questioning by a health professional, 8/44 patients (18%) reported symptoms that might suggest OSA including snoring, mouth breathing and restless sleep.

**Table 1:** Demographic and Anthropometric Data in Children with PWS.

	All patients n = 44	< 2 years n = 23	≥2 years n = 21	p
**Median Age (y)**	1.9 [0.3, 15.6]	1.0 [0.3, 1.9]	5.1 [2.4,15.6]	-
**Females**	24/44 (55%)	11/23 (48%)	13/21 (62%)	0.349
**Mean BMI z-score**	1.3±2.1	0.1±1.3	2.6±2.1	<0.001
**Obese and overweight**	23/44 (52%)	6/23 (26%)	17/21 (81%)	<0.001

BMI- body mass index. Continuous data are expressed as median (range) or mean ± standard deviation. Categorical variables are expressed as frequencies and proportions.

### Polysomnography Results

**Figure 1:**
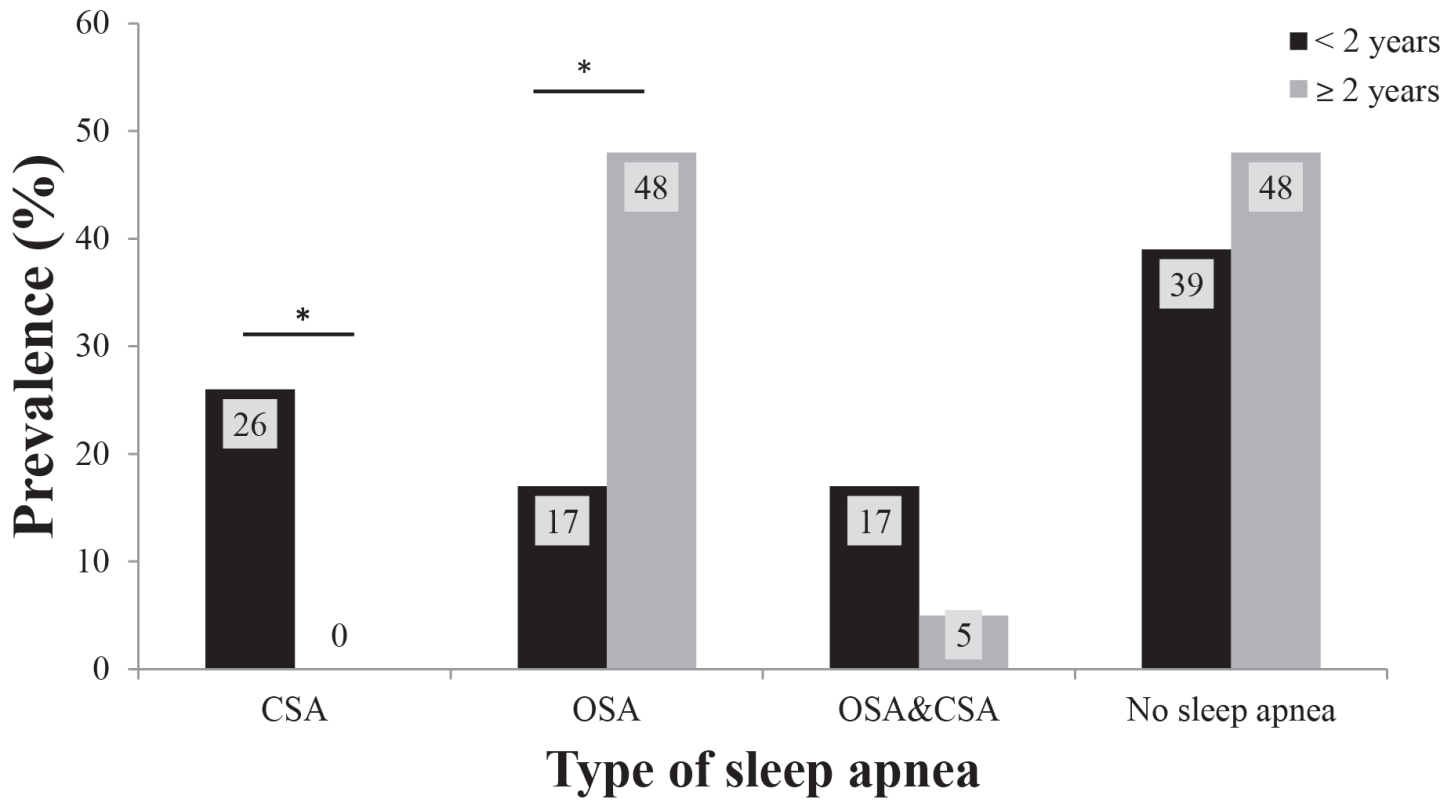
Prevalence of sleep apnea stratified by age group. Below the figure: CSA- central sleep apnea; OSA- obstructive sleep apnea; *- p<0.05.

SDB was common in both infants and older children (Table 2). Overall, 8/44 patients reported symptoms that may be associated with sleep apnea, and sleep apnea was diagnosed on a PSG in 25/44 (57%) patients. Of the eight patients for which parents reported symptoms, 7 had sleep apnea on the PSG. Specifically, one patient had CSA, 3 patients had OSA and 3 patients had both OSA and CSA. The eighth patient was not diagnosed with sleep apnea on a PSG. Eleven out of 44 patients (25%) had CSA including either CSA alone [n = 6] or both CSA and OSA [n = 5]; ten were infants and one was >2 years of age. The median CAI in patients with CSA was 10.6 events/hour (range 5.0–68.3). The median OAHI in those with OSA was 4.0 events/hour (range 1.5–57.0). Significant differences in the prevalence of sleep apnea were observed between the 2 groups (Figure 1, overall p = 0.016). Compared with older children, infants were more likely to have CSA (43% vs. 5%; p = 0.003) and median SpO_2_ nadir trended to be lower in infants (80% (range 52, 93) vs. 88% (range 27, 95); p = 0.094). OSA were non-significantly less prevalent in infants when compared with older children (35% vs. 52%; p = 0.239). Interestingly, occurrence of OSA alone (not co-existent OSA and CSA) was significantly less frequent in infants compared with older children (17% vs. 48%; p = 0.032). While in infants there was no significant difference between the occurrence of OSA and CSA (35% vs. 43% respectively, p = 0.546), in older children OSA clearly predominated (52% vs. 5% p = 0.001).

**Table 2:** Polysomnography Study Data in Children with PWS.

PSG results	All patients n = 44	<2 years n = 23	≥2 years n = 21
**OSA**	14/44 (32%)	4/23 (17%)	10/21 (48%)
**CSA**	6/44 (14%)	6/23 (26%)	0/21 (0%)
**OSA&CSA**	5/44 (11%)	4/23 (17%)	1/21 (5%)
**Sleep efficiency (%)**	90 [59, 100]	90 [59, 98]	88 [73,100]
**Sleep latency (min)**	10 [0, 91]	17.0 [0, 91]	9 [0, 52]
**REM latency (min)**	75.3 [0.4, 180]	53.1 [0.4, 180]	95.4 [11,172.5]
**SpO_2_ nadir (%)**	83 [27, 95]	80 [52, 93]	88 [27, 95]

CSA- central sleep apnea, OSA- obstructive sleep apnea, REM-rapid eye movement, SpO_2_- peripheral oxygen saturation. Continuous data are expressed as median (range). Categorical variables are expressed as frequencies and proportions.

The mean total sleep time recorded was 491±50 minutes (8.2±0.8 hours) and the mean total sleep time for the 4 split night studies was 445±88 minutes (7.4±1.5 hours). Sleep architecture including sleep efficiency, sleep latency and the percent of time spent in the different sleep stages were similar to normal values reported in the literature for children without PWS [9], [19]–[21]. The median percent time spent in N1, N2, N3 and REM in infants was 3% [0, 15], 34% [22, 55], 35% [11, 59] and 28% [13], [35] respectively. The median percent time spent in N1, N2, N3 and REM in the older children was 5% [1, 67], 50% [9, 66], 20% [0, 36] and 19% [0, 32] respectively. As reported by others, REM latency appeared to be shorter than normal; median REM latency was 53.1 minutes [0.4, 180] in infants and 95.4 minutes [11, 172.5] in the older children.

### Oxygen treatment for CSA in infants

Of the 11 PWS patients with CSA (CAI ≥5.0 events/hour and SpO_2_ nadir <92%), 9 infants were treated with nocturnal supplemental oxygen for (Table 3). Two patients with CSA were not treated with supplemental oxygen, both had CSA and co-existing OSA. One was a 1.7 years old infant that was lost to follow-up for over a year after the initial PSG. The second patient was a 2.9 year old young child with significant severe OSA, OAHI of 57 events/hour as well as a CAI of 9.7/hour. He was referred to Otolaryngology for an adenotonsillectomy for management of his severe OSA. In the patients treated with oxygen, supplemental oxygen flow ranged between 0.25–1.0 L/min, the lowest value providing beneficial results was recommended. Four infants had a single split night PSG study and 5 infants had 2 full PSGs; in these cases the time between the 2 PSGs ranged between 28–72 days. The median CAI and SpO_2_ nadir prior to oxygen therapy was 14 events/hour (range 5, 68) and 70% (range 52,92) respectively. The median number of desaturations of 3% or more per hour (Desaturation index [DI]) was 15.2 (range 1.5, 70). The majority of central apneas were associated with oxygen desaturations. Following oxygen therapy, there was a significant decrease in CAI to a median of 1 event/hour (range 0, 6; p = 0.008) and a trend towards significant improvement in the SpO_2_ nadir, from 70% to 82% (range 64, 95; p = 0.080). Of particular relevance, with application of oxygen, the majority of the central events were eradicated in addition to an improvement in SpO_2_. There was no corresponding hypercapnic response with oxygen treatment; maximum tcCO_2_ (mmHg) was 47 (range 44–51 mmHg) on room air, and 45 (range 36–53 mmHg) with supplemental oxygen (p = 0.121).

**Table 3:** Age, Anthropometrics and Polysomnography results for infants treated with supplemental oxygen for CSA.

Patient	Room Air								Supplemental Oxygen					
	Study age (y)	length z-score	BMI z-score	CAI	OAHI	SpO_2_ nadir	DI	tcCO_2_ Max	Study age (y)	O_2_ (L/min)	CAI	OAHI	SpO_2_ nadir	tcCO_2_ Max
1*	0.4	−2.9	−0.3	5	0.8	70	6.3	49	0.4	0.3	0	0.4	95	44
2*	0.3	−0.3	−1.7	5.8	4.1	92	1.5	n/a	0.3	0.4	2	5	77	n/a
3	0.6	−4.6	1.2	68.3	5.6	52	70	51	0.7	0.5	5.1	0	76	46
4*	0.7	−0.4	−1.2	26.9	0	66	20.9	44	0.7	0.3	1	0	81	36
5	1	−2.1	−1.5	6.8	0.3	84	4.9	44	1.2	0.25	0.3	0.4	95	40
6	0.6	−3.1	−1.3	13.7	1.7	70	15.2	47	0.7	0.5	0.5	3.2	83	53
7	1.2	−2.2	−0.3	10.6	0.5	73	9.2	47	1.3	0.8	3.6	0.9	84.2	46
8	0.5	−2.4	1.1	23.3	11.6	61	23.5	44	0.5	1.0	0.9	22.2	64	39
9*	0.3	−3.88	−0.51	13.7	2.8	76	16.8	47	0.3	0.25	6.1	6	76	46

Polysomnography results on room air and with supplemental oxygen are presented.

BMI-body mass index; CAI-central apnea index; OAHI- obstructive apnea hypopnea index; SpO_2_- saturation of peripheral oxygen; tcCO_2_- trans cutaneous carbon dioxide; O_2_- oxygen; *- split night study, DI number of desaturations >3% per hour; n/a- not available.

## Discussion

To our knowledge this is the first analysis stratifying patterns of sleep disordered breathing in infants and older children with PWS. The principal findings of this study were that 1) CSA (defined as a CAI ≥5 with associated oxygen desaturations) was common in infants with PWS 2) CSA was effectively treated with supplemental oxygen and 3) CSA was uncommon beyond 2 years of age when OSA predominated. The strength of this study is that it included many infants and children with PWS who were GH naïve and had undergone full PSGs. Specifically, these patients were not selected for symptoms or for management of a specific type of SDB.

Traditionally, PWS has been associated with OSA due to co-existing obesity, narrowing of the upper airways and respiratory muscle hypotonia [22]–[24]. Although OSA was observed in 35% of infants, this was mild in the majority of cases and no infant required an adenotonsillectomy. Most infants with PWS are not obese, many are in fact underweight for age, yet increased fat mass has been demonstrated even in this age group [25], [26]. Combined with the significant hypotonia and narrower airway diameter this could contribute to the occurrence of OSA at younger ages. An interesting finding was the high number of infants with CSA and associated significant oxygen desaturations. The etiology of CSA in PWS infants is thought to be multifactorial. Contributing mechanisms include hypotonia, brainstem immaturity and hypothalamic dysfunction [24]. Abnormal chemosensitivity to CO_2_ and O_2_ may contribute to hypoxia induced respiratory depression.

The primary treatment strategy for CSA and central hypoventilation syndromes is positive airway pressure. An important finding in this study was that low flow oxygen not only improved oxygen saturations but effectively eradicated central apneas obviating the need for positive pressure ventilation in these patients. The biological mechanisms of the therapeutic effects of oxygen on central apneas is unclear. Since PWS subjects have abnormal chemosensitivity to both hypoxia and hypercapnia, it is likely that even small degrees of hypoxia may have a destabilizing effect on the control of breathing, resulting in central depression. Low flow oxygen may stabilize the control of breathing, preventing hypoxia induced respiratory depression.

Our results confirm the findings of a recent case series evaluating the use of supplemental oxygen treatment in infants with PWS [10]. However, that series included a selected sample of infants with a known diagnosis of CSA treated with oxygen. Additionally, information regarding the frequency of CSA or other SDB in PWS infants not treated with oxygen was not shown. An interesting observation was that the PWS infants in the case series demonstrated milder CSA than in the current study. It is not clear whether any of the infants were treated with GH at the time of the PSG, a treatment that might affect CSA severity.

The literature supports a high prevalence of SDB in pediatric PWS [4], [27]–[29]; however no study has directly compared SDB in infants with older children to systematically test the magnitude of differences [8], [9], [30]. Of note a study of 34 infants and children with PWS found the mean age of those with OSA to be significantly higher when compared to those without OSA (9.8±4.6 vs. 5.3±4.8 years) [31]. In one study of infants with PWS prior to and following GH treatment initiation, the total number of central events during the night ranged from 0–84, however the total sleep time was not provided. Direct comparisons with our current data were limited as the number of central apnea events per hour could not be determined. However, it is likely that several infants within that cohort did have an abnormal CAI with associated oxygen desaturations [8]. Our study adhered strictly to the AASM diagnostic criteria for sleep associated events [12], comparison with some studies is difficult due to the varying diagnostic criteria implemented for CSA [9], [30]. Additionally, the inclusion of patients already treated with GH or with a diagnosis of SDB might have biased results in some studies [4], [10], [29]. Little is known regarding age-related patterns of SDB in PWS, particularly from infancy onward. One study evaluated children and adults with PWS and did not detect persistent longitudinal trends [29]; however of significance, infants <2 years of age were not included. Importantly 43% of the study population did not experience sleep apnea. Data on prevalence of sleep apnea in the PWS population vary greatly [24], prevalence is reported to range from zero in older children and adults [32] to 100% in infant subjects with PWS [30]. This variability is at least partly explained by differences in the specific characteristics of the sleep apnea [28]. Variable and wide age ranges, varying diagnostic criteria and a potential referral bias may contribute to differences in SDB prevalence estimations between studies.

Infants with PWS are increasingly referred for GH treatment, prior to which PSG screening is recommended. The early use of GH is likely related to the early diagnosis of PWS and to recent studies suggesting benefits for treatment in infancy [33]. Obesity related OSA remains the major focus when discussing SDB in pediatric PWS [34] and routine and follow-up PSG screening is recommended in consensus guidelines and expert reviews mostly in the context of GH treatment [1], [18], [35]. Specific emphasis on SDB in infants with PWS is lacking particularly with regards to CSA. This might be related to the aforementioned limitations of the available studies, as well as to the lack of data regarding treatment of CSA.

The clinical importance of the findings of a high prevalence of CSA with associated oxygen desaturations in infancy requires further consideration. It is well documented that OSA is associated with poorer neurocognitive outcomes in children with and without PWS [30], [35]–[38]. Importantly, it is hypothesized that intermittent hypoxia is associated with oxidative stress and inflammation which may promote end-organ dysfunction [39]. To date, as CSA disorders are uncommon in children, the long term consequences of CSA are unknown. If indeed CSA and hypoxemia in infancy are associated with long term neurocognitive deficits, recognition and treatment early in infancy may have particularly beneficial outcomes in this at-risk population.

The limitations of our study include the fact that PSG studies were only performed on patients considered for GH therapy. However, currently, most infants with PWS are being referred for consideration of GH therapy regardless of clinical characteristics. Another limitation of our study is that a control group of healthy infants and children was not available for comparison. Lastly, we performed split-night studies in 4/9 of the infants with CSA, thus observing sleep for shorter though reasonably representative periods on each part of the night when compared with full night studies. Based on our experience, it is part of our current practice to perform a “split night” PSG study particularly for families who are unable to attend for 2 nights. That is, infants demonstrating significant CSA while in room air in the first half of the night, are treated with supplemental oxygen in the second half. This approach allows earlier management of CSA, particularly if PSGs are not widely available. It obviates the need for a second PSG, sparing the child and family the discomfort involved in performing an additional PSG with an overall reduction in costs.

In conclusion, CSA was found to be prevalent in infants with PWS. Importantly, infants with a CAI ≥5 events per hour with associated oxygen desaturations were effectively treated with low flow oxygen. After 2 years of age, OSA predominated in PWS patients. How CSA and related hypoxemia may impact the developing brain in this vulnerable population is unknown. Oxygen therapy may improve long-term neurocognition and only longitudinal studies would be able to establish the benefit of oxygen in PWS infants. Until further conclusive evidence suggests otherwise, minimizing nocturnal oxygen desaturations and treating CSA should be considered in all infants with PWS. The authors recommend that all children with PWS, with particular attention to infants, be screened for SDB irrespective of plans for GH therapy. Until further conclusive evidence suggests otherwise, treating CSA with associated nocturnal oxygen desaturations should be considered in all infants with PWS.
